# Inflammation and Infection in Critical Care Medicine

**DOI:** 10.1155/2014/456256

**Published:** 2014-02-02

**Authors:** Jesús F. Bermejo-Martin, Ignacio Martín-Loeches, Steven Bosinger

**Affiliations:** ^1^Laboratorio de Ciencias Biomédicas en Investigación Clínica (CBIC), Hospital Clínico Universitario de Valladolid, IECSCYL-SACYL, Avenida Ramón y Cajal 3, 47005 Valladolid, Spain; ^2^Critical Care Centre, Corporació Sanitària i Universitaria Parc Taulí—Hospital de Sabadell, Institut Universitari UAB Ciber Enfermedades Respiratorias, Parc Taulí 1, Sabadell, 08208 Barcelona, Spain; ^3^Department of Microbiology & Immunology, Yerkes National Primate Research Center, Emory University, Rm 3028 Emory Vaccine Center Building 954, Gatewood Road NE, Atlanta, GA 30329, USA

Inflammation and infection are closely linked in critically ill patients. When adaptive immunity fails to prevent or control infection, an exacerbated/maintained inflammatory response emerges as consequence. It could be considered as an attempt of the immune system to fight the infection, which, in so many occasions, results insufficiently. In turn, uncontrolled inflammatory responses impair the development of specific and targeted responses against the infecting microbe, closing a vicious circle. The consequences of unbalanced inflammatory and adaptive responses to infection are microbial escape and tissue damage, contributing to the physiopathogenesis of sepsis, acute respiratory distress syndrome, or multiorganic failure. In this special issue, Steel C. et al. review the major virulence factors of *S. pneumoniae* and their role in triggering overexuberant inflammatory responses contributing to the immunopathogenesis of invasive disease in community-acquired pneumonia (CAP). These authors provided an insight into the pharmacological, anti-inflammatory strategies with adjunctive potential in the antimicrobial chemotherapy of CAP. In turn, Surbatovic M. et al. explored some of the more novel elements of immunoinflammatory response in severe sepsis and severe trauma, highlighting the role of Toll-like receptors, cytokines, and the genetic polymorphisms in the immune response to infection. Esteban E. et al. summarized the immune modulation actions of extracorporeal devices in the context of endotoxin removal, elimination of cytokines and inflammatory molecules, vascular and coagulation proteins, and removal of cells, which might play a role as an innovative coadjuvant treatment in sepsis or nonseptic respiratory failure. De Pascale G. et al. reviewed the role of mannose-binding lectin (MBL) in severe sepsis and septic shock. MBLs are serum proteins that recognize a wide range of pathogenic microorganisms and activate complement cascade via the antibody-independent pathway. While MBL-deficient patients are at increased risk of infection acquisition, an excess of MBL activation may drive unbalanced proinflammatory responses and additional host injury. MBL replacement therapy was also discussed. An original article from Rodriguez-Fernandez A. and colleagues revealed the protective role of eosinophils in *S. aureus* ventilator-associated pneumonia. Being a recognised actor in the pathophysiology of asthma, there is an increasing body of evidence on the antimicrobial roles played by eosinophils. This paper highlights the potential of an unexpensive tool such as the leukogram as biomarker in severe infections. Finally, Koch A. et al. investigated the regulation and prognostic relevance of symmetric dimethylarginine (SDMA) serum concentrations in critical illness and sepsis. SDMA impacts vascular tension and integrity via modulating nitric oxide (NO) pathways. These authors identified SDMA serum concentrations at admission as an independent prognostic biomarker in critically ill patients not only for short-term mortality at the ICU but also for unfavourable long-term survival, with patients with sepsis showing the highest SDMA levels. They proposed this molecule as a novel biomarker for mortality risk stratification for ICU patients.

We hope that this compilation will be of interest for both clinicians and researchers working in the field of host immune responses to infection in severely ill patients.

*Jesús F. Bermejo-Martin* *Jesús F. Bermejo-Martin* *Ignacio Martín-Loeches* *Ignacio Martín-Loeches* *Steven Bosinger* *Steven Bosinger*

